# Microgravity and the intervertebral disc: The impact of space conditions on the biomechanics of the spine

**DOI:** 10.3389/fphys.2023.1124991

**Published:** 2023-03-14

**Authors:** Giovanni Marfia, Laura Guarnaccia, Stefania Elena Navone, Antonella Ampollini, Melissa Balsamo, Francesca Benelli, Chiara Gaudino, Emanuele Garzia, Claudia Fratocchi, Claudia Di Murro, Gianfranco Kim Ligarotti, Carmelo Campanella, Angelo Landolfi, Pietro Perelli, Marco Locatelli, Giuseppe Ciniglio Appiani

**Affiliations:** ^1^ Laboratory of Experimental Neurosurgery and Cell Therapy, Neurosurgery Unit, Fondazione IRCCS Ca’ Granda Ospedale Maggiore Policlinico, Milan, Italy; ^2^ Clinical Pathology Unit, Istituto di Medicina Aerospaziale “A. Mosso”, Aeronautica Militare, Milan, Italy; ^3^ Department of Neuroradiology, Azienda Ospedaliero-Universitaria Policlinico Umberto I, Rome, Italy; ^4^ Istituto di Medicina Aerospaziale “A. Mosso”, Aeronautica Militare, Milan, Italy; ^5^ Istituto di Medicina Aerospaziale “Aldo Di Loreto”, Aeronautica Militare, Rome, Italy; ^6^ Italian Air Force Logistic Command, Rome, Italy; ^7^ Department of Medical-Surgical Physiopathology and Transplantation, University of Milan, Milan, Italy

**Keywords:** microgravity, intervertebral disc, disc degeneration, low back pain, space

## Abstract

The environmental conditions to which astronauts and other military pilots are subjected represent a unique example for understanding and studying the biomechanical events that regulate the functioning of the human body. In particular, microgravity has shown a significant impact on various biological systems, such as the cardiovascular system, immune system, endocrine system, and, last but not least, musculoskeletal system. Among the potential risks of flying, low back pain (LBP) has a high incidence among astronauts and military pilots, and it is often associated with intervertebral disc degeneration events. The mechanisms of degeneration determine the loss of structural and functional integrity and are accompanied by the aberrant production of pro-inflammatory mediators that exacerbate the degenerative environment, contributing to the onset of pain. In the present work, the mechanisms of disc degeneration, the conditions of microgravity, and their association have been discussed in order to identify possible molecular mechanisms underlying disc degeneration and the related clinical manifestations in order to develop a model of prevention to maintain health and performance of air- and space-travelers. The focus on microgravity also allows the development of new proofs of concept with potential therapeutic implications.

## 1 Introduction

Atmospheric flight, especially spaceflight, represents a unique “exposome” that exposes pilots, astronauts, and space crew to peculiar conditions such as ionizing radiation, microgravity, pressure variations, absence of circadian rhythm, and noise pollution. One of the interesting aspects concerning the incidence of low back pain (LBP) among pilots and astronauts is that it is certainly related to multiple aspects, such as posture and vibration, as well as exposure to microgravity.

LBP is one of the top three causes of disability in developed countries (Murray et al., 2012). An analysis conducted by the 2021 Global Burden of LBP shows that in 2017, the prevalence of LBP was about 7.5% of the world population (Wu et al., 2020). Over the years, the disability associated with this condition has increased, and the most affected age group is 50–54 years (2019). These data have a serious/important socioeconomic impact because 70% of the years of work lost involve people of the working age group (20–65) (Owen and Williamson, 2021).

Although the etiology of LBP is not fully understood and different anatomical structures may contribute to the onset of pain, it is typically associated with intervertebral disc (IVD) degeneration (Baliga et al., 2015). IVD degeneration (IDD) is considered an aberrant, pathological, cell-mediated response that leads to progressive structural damage of the IVD with pain (Adams et al., 2015). Nowadays, therapeutic strategies for treating IDD and alleviating related pain include conservative treatments, such as anti-inflammatory drugs. When these treatments are ineffective, radical surgery (e.g., disc removal and spinal fusion) is considered, although its purpose is not to restore the physiological structure and biomechanical properties of the disc but only to remove the source of pain.

Several studies have demonstrated the impact of spaceflight conditions, and particularly microgravity, on the musculoskeletal system, including IVD and its mechanisms of degeneration. In the following sections, we discuss the mechanisms of IDD, the microgravity conditions to which astronauts are subjected, and the experimental evidence confirming their association. Methods for simulating microgravity, both in orbit and on Earth, have also been described in order to consider the application of microgravity as a therapeutic approach for degenerative spinal pathologies and to suggest the potential for applying early, individualized countermeasures that avoid not only the onset of painful symptomatology but also its impact on work capacity and aerospace medicine.

## 2 The intervertebral disc

IVD is a complex, heterogeneous, and specialized structure consisting of fibrocartilaginous connective tissue positioned between two adjacent vertebrae. The IVD functions to impart limited flexibility to the body trunk, provide mechanical stability during axial compression and movement, and protect both the spinal cord and spinal nerves (Navone et al., 2017).

The main purpose of the IVD is to allow articulation between vertebrae by preventing friction between them. The IVD accounts for 20%–30% of the length of the spine, which plays a key role in protecting the bone marrow from injury, weight dispersion, movement, and nutrient transport (Raj, 2008). The healthy IVD consists of three structurally distinct and interdependent components: a gelatinous nucleus called the nucleus pulposus (NP), a lamellar outer ring of fibrous tissue called the annulus fibrosus (AF) surrounding the NP, and two cartilaginous endplates (CEPs) that serve as the interface between the disc and the vertebrae by covering both the NP and the AF cranially and caudally (Raj, 2008).

The main component of the inner and outer rings is water; the other components include collagen (type I and type II), proteoglycans, and other extracellular matrix proteins. The composition of the extracellular matrix is variable: moving away from the NP increases the presence of type I collagen (at the expense of type II) and the amount of proteoglycans decreases (Guerin and Elliott, 2007). In the fibrous ring, collagen is responsible for the structural and mechanical appearance and typical ring shape (Smith and Fazzalari, 2009). The NP forms the central part of the IVD. It consists of proteoglycan hydrogels in which there are randomly organized fibers of type II collagen and elastin (Perie et al., 2006). The central part of the NP is mainly composed of water, which enables the IVD to play its role of shock absorption and load dispersion.

The healthy adult IVD is almost entirely avascular, and only specialized capillaries, between the bone endplate and the CEPs, provide a limited supply of nutrients and oxygen that reach the interior of the IVD by passive diffusion through the CEPs (Urban et al., 2004). In addition, the healthy IVD is generally considered a sparsely innervated organ. Innervation, normally but not exclusively accompanied by vascularization, is restricted to the outer layers of the AF and consists of perivascular sensory and sympathetic nerve fibers. In particular, sensory fibers innervating the IVD have been shown to be small sensory nerves (both peptidergic and non-peptidergic nociceptors) as well as larger fibers forming proprioceptors.

## 3 IVD degeneration

Although the precise etiology of IDD remains unclear, degeneration occurs as a natural aging event, and numerous studies have shown that this process can be potentially accelerated or exacerbated by the synergistic contribution of environmental and genetic factors (Virtanen et al., 2007; Wang et al., 2016a; Wang et al., 2016b). Several studies on IDD have highlighted three likely triggers: biomechanical wear, lack of nutritional factors, and presence of pathogens (Urban et al., 2004; Alpantaki et al., 2011; Adams et al., 2015). As for environmental risk factors, an unhealthy lifestyle, for example, lack of exercise (Elfering et al., 2002), smoking (Cong et al., 2010; Wang et al., 2012), mechanical influences and occupational exposures (e.g., heavy lifting (DePalma et al., 2012), vibration (Virtanen et al., 2007), trauma (Heyn et al., 2014; Schroeder et al., 2014), and infectious agents (Stirling et al., 2001), has been suggested as a contributing cause of IDD. Furthermore, the importance of genetic factors in the development of IDD has become evident in recent years because of studies that have identified a correlation between polymorphisms in many key genes (Navone et al., 2012), such as the collagen I gene COL1A1 (Tilkeridis et al., 2005; Videman et al., 2009), the vitamin D receptor gene (Videman et al., 1998; Kawaguchi et al., 2002), several protease genes related to matrix degradation (Zhang et al., 2013; Zawilla et al., 2014), and different degrees of IDD (Kepler et al., 2013a; Kepler et al., 2013b), as well as the existence of a familial predisposition to IDD (Kalichman and Hunter, 2008; Battié et al., 2009). During the early stages of IDD, the main detectable features are the onset of an inflammatory condition in disc tissues, gradual degradation of NP and AF, and reduced viability of resistant cells (Navone et al., 2021). These pathological changes may subsequently lead to aberrant innervation and vascularization of the IVD and collapse of the spinal motion segment through NP herniation outside the AF or complete degradation of the NP within an intact AF (such as the “black disc”). All these events can compromise the entire functional properties of the spine and consequently cause pain (Smith et al., 2011; Kepler et al., 2013b).

**FIGURE 1:**
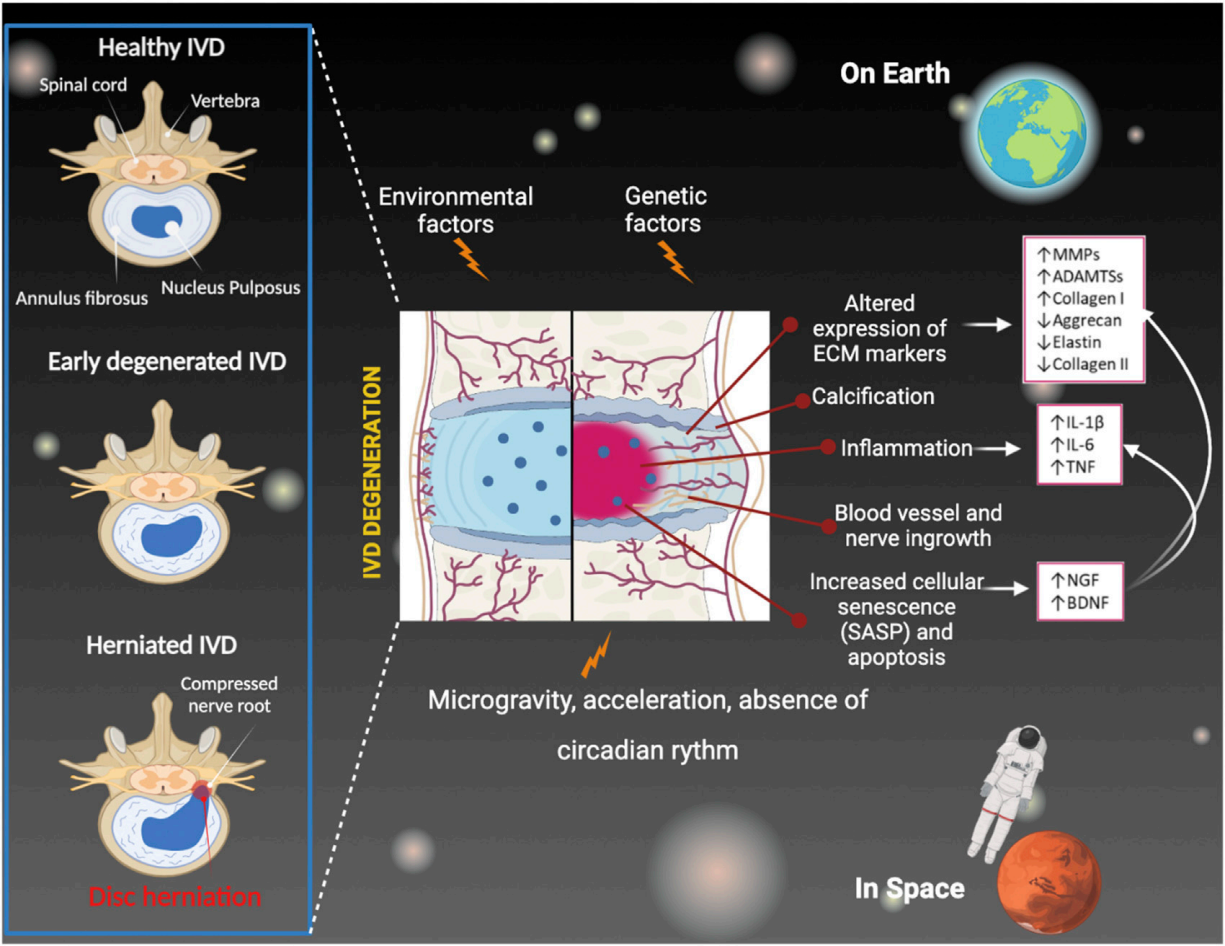
IVD degeneration mechanisms under Earth’s conditions and during spaceflight and missions.

IDD is characterized by the early onset of a severe inflammatory environment both within the degenerating IVD and in the peridiscal space, followed by the production and secretion of pro-inflammatory factors (e.g., cytokines) such as interleukin 1b (IL-1b), interleukin 6 (IL-6), and tumor necrosis factor α (TNF-α) (Takahashi et al., 1996; Shamji et al., 2010). These inflammatory mediators are produced by resident IVD cells as well as by circulating immune cells that infiltrate within the IVD (which, under physiological conditions, does not contain a resident immune cell population) due to the favorable conditions generated during IDD. Specifically, in the degenerating IVD, the infiltration of activated immunocytes, including macrophages, T and B cells, and natural killer (NK) cells (Geiss et al., 2007; Kokubo et al., 2008; Murai et al., 2010; Shamji et al., 2010), occurs in response to the expression of several chemokines by IVD cells and is enabled by the loss of the structural integrity of the extracellular matrix (ECM) of the disc (Navone et al., 2018). It has been shown that the onset of this severe inflammatory environment within the degenerating IVD triggers a series of pathogenic responses, such as ECM degradation, internal nerve damage, and vascular growth, which eventually lead to massive degeneration and can cause pain (Takahashi et al., 1996; Burke et al., 2002; Shamji et al., 2010). Furthermore, it has been shown that the increasing number of senescent NP cells plays an important role in the initiation of IDD. This effect is particularly due to the secretion of metabolic factors, collectively known as the senescence-associated secretory phenotype (SASP), as chemokines, growth factors, pro-inflammatory cytokines, and matrix-degrading proteases, which contribute to the ECM modifications (Gruber et al., 2011; Zhao et al., 2011; Bedore et al., 2014). In recent years, this finding prompted the potentiality to optimize treatments reducing senescence to slow down IDD and relieve LPB (Ngo et al., 2017) (Figure 1).

## 4 Real and simulated microgravity: What is its contribution to IDD?

Microgravity has demonstrated a significant impact on important structural and functional properties of cells, including cell morphology, proliferation, and migration (Bradbury et al., 2020) in both healthy and pathological cellular models (e.g., cancer cells), so much that gravitational biology has become a hot topic in space research and aerospace medicine.

Gravity determines almost all physical, chemical, and biological phenomena that occur on our planet. Everything on Earth is subjected to gravity, and a person’s weight corresponds to the force exerted on the mass of the human body by Earth’s gravitational field. The effect of gravity on an object can be completely nullified when it experiences “free fall,” as it does in orbit. This state is due to microgravity, which refers to an environment where gravity is less than that found on Earth’s surface, and is commonly called weightlessness. The physical and biological adaptive changes that occur during space missions highlight the importance of gravity during human evolution and an association between microgravity, aging, and disease onset. In space, this lack of gravity causes the loss of mechanical stimulation of cells and tissues and is therefore responsible for many of the physiological problems that astronauts experience, including diseases of the musculoskeletal system.

Under normal gravity conditions, the spine is subjected to diurnal changes in height and hydration. When we are standing, the spine is upright so that gravity compresses the discs, expelling water. During the day, the height of the disc decreases, the curvature of the spine changes, and the spine becomes more flexible. During sleep, in a horizontal position, the gravity load is lost so that the discs rehydrate, absorb water, and swell. This turnover allows the disc to regain physiological height and water content, maintaining structural and functional alignment. In space, the loss of diurnal fluctuation and microgravity results in an imbalance, as gravity is unable to counteract the discs’ inherent propensity to attract water (Adams et al., 1987).

Microgravity research is essential to reveal the impact of gravity on biological processes and organisms. Spaceflights to the International Space Station (ISS) provide unique conditions to study microgravity. However, research on near-Earth orbit is severely constrained by the limited number of flight opportunities, as experiments would have to be performed autonomously, the design is reasonably difficult, costs are higher than other flight options, and preparation takes years (Prasad et al., 2020).

For short periods, real microgravity can be experienced using instruments such as the Bremen Drop Tower, Germany, where an airtight capsule is dropped into an evacuated tube inside the tower. It represents a unique facility in Europe for performing experiments under weightless conditions with residual gravitational accelerations in the microgravity regime (Messerotti Benvenuti et al., 2011; Acharya et al., 2017). The maximum period of 4.74 s of each free-fall experiment at the Bremen Drop Tower is limited only by the height of the drop tower vacuum tube, which is completely fabricated by steel and enclosed by a concrete outer shell.

**FIGURE 2:**
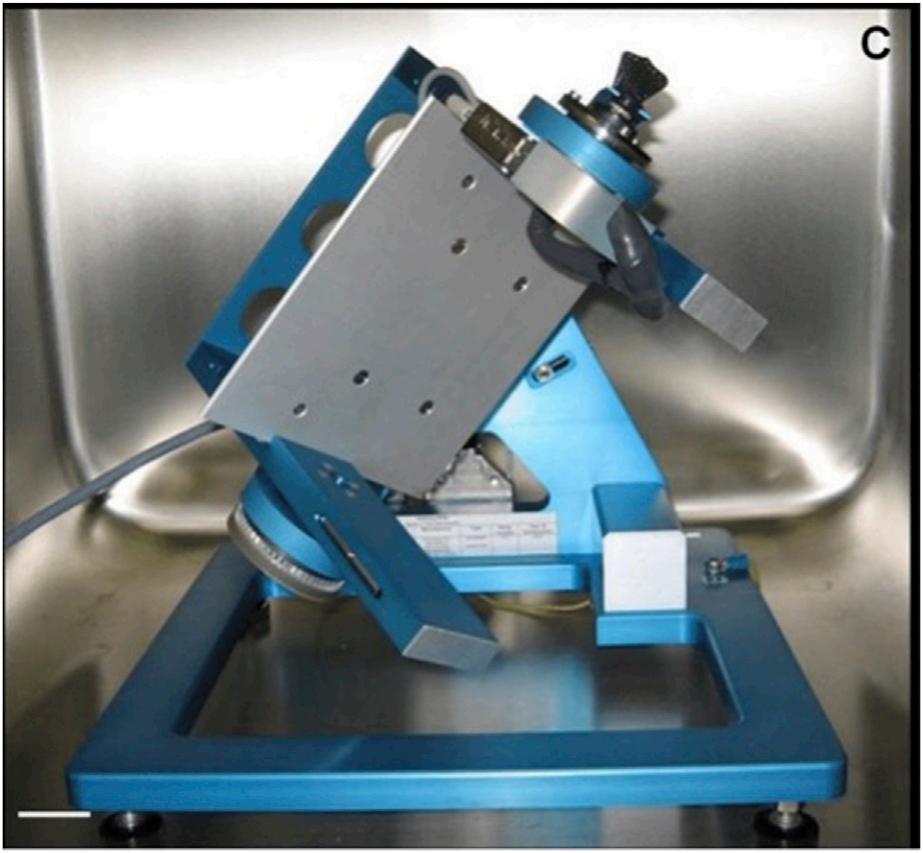
Random positioning machine (Kopp, 2011).

Parabolic flights are used for longer microgravity exposure due to the aircraft’s flight angle creating a microgravity condition that lasts about 22 s and is surrounded by two hypergravity phases with about 2 G for 20 s each (Acharya et al., 2017). An even longer microgravity exposure can be achieved by suborbital flight with a sounding rocket, allowing 6 min (TEXUS) or 13 min (MAXUS/MAPHEUS) of microgravity. On Earth, techniques for studying the impact of microgravity on the human body include the “head-down bed rest” (HDBR) method, in which the subject lies on a bed with the head tilted 6° (Messerotti Benvenuti et al., 2011). This condition can be used for short-term investigations (e.g., 72 h) (Liao et al., 2012) or long-term studies (e.g., 90 days) (Roberts et al., 2010), which mimic many effects of spaceflight on the human body, such as a decrease in bone density, muscle mass, and cephalic fluid strength and displacement. In parallel, the most reliable device currently available for testing simulated microgravity on *in vitro* cellular models is the 3D-clinostat, also called the random positioning machine (RPM). The 3D-clinostat is a multidirectional G-force generator, consisting of a central platform in which a housing for positioning the biological sample is attached, interconnected with two perpendicular arms that rotate independently, thus providing continuous rotation with two axes (Becker and Souza, 2013). In this way, the 3D-clinostat cancels the gravity vectors at the center of the device, allowing the cell inside to experience a microgravity environment averaging 10–3 G over time, due to which a lack of sedimentation and the growth of 3D multicellular spheroids can be observed (Figure 2). The continuous rotation of the RPM provides constant randomization of the gravity vector, making this device a useful adjunct to prepare for spaceflight studies. Notably, this on-ground model may present some limitations, as it has been recently reported that it may produce false‐positive results that could be misinterpreted as cellular microgravity responses. For example, Mansour et al. (2023) described an impairment of myotube formation in murine myoblasts (C2C12 cells) cultured in a 2D-clinostat as an apparent effect of microgravity. However, focused experiments proved that this event was equally a consequence of fluid motion, suggesting that biological results from cellular models must be rigorously tested and ruled out before being attributed to microgravity.

A specialized form of clinostatism is the rotating wall vessel (RWV) bioreactor (Schwarz et al., 1992), developed by NASA, which consists of a horizontally rotating vessel without an internal mechanical stirrer, in which the vessel provides an environment characterized by low turbulence and shear.

An alternative method to simulate microgravity is immersion. The subject can be immersed either horizontally or vertically, depending on whether immersion occurs completely or not. The depth and temperature can also be variable. Immersion is called “dry” when the subject is isolated from the aqueous medium by impermeable fabrics. In this case, immersion can last up to days. As for “wet” dives, these occur without any kind of insulation and cannot last more than a couple of hours (Navasiolava et al., 2011).

## 5 Effects of microgravity on disc degeneration

The main experimental evidence obtained through *in vitro* studies on cellular models and *in vivo* studies on animal models and volunteer astronauts has been discussed in the following section. Table 1 shows the results of the selected studies which have been discussed later.

**TABLE 1:** Summary of selected articles addressing research on the effects of simulated microgravity on the intervertebral disc.

Reference	Object	Method	Result
Messerotti Benvenuti et al. (2011)	Learning	A total of 22 male matching astronauts were divided into two groups	Habituation analysis showed a normal reflex inhibition across blocks in sitting controls and no habituation in HDBR subjects
	Brain plasticity	1) Head-down bed rest (HDBR)	
		2) Sitting control	
		EMG of the orbicularis oculi muscle used to measure startle reflex amplitude during picture viewing with acoustic startle probes	
Roberts et al. (2010)	Corticospinal plasticity	Four subjects underwent functional MRI (fMRI), transcranial magnetic stimulation (TMS), and functional mobility testing (FMT) before and after 90 d of bed rest	- Leg recruitment curve slope decreases
			- Hand recruitment curve slope increases
			- No change in activation for the hand but an increase in activation post-bed rest for the leg
Bailey et al. (2014)	Biomechanical properties of lumbar and caudal discs	Eight C57BL/C mice on 15-day mission aboard STS-131	- Caudal discs: the spaceflight group—32% PDH; 70% D
		- Physiological disc height (PDH) measurement in situ	- Lumbar discs: no difference in PDH and D
		- Compressive creep tests to parameterize:	- k and G for lumbar and caudal discs did not appear to be influenced by microgravity
		1) Endplate permeability (k)	
		2) Nuclear swelling pressure strain dependence (D)	
		3) Annular viscoelasticity (G)	
Wu et al. (2017)	Molecular biological changes in IDD under weightlessness and hypergravity conditions	A total of 120 rabbits were divided into control, weightlessness, hypergravity, and mixed (hypergravity + weightlessness) groups, simulated by tail-suspension and animal centrifuge	- MMP-1, MMP-3, and TIMP-1 were expressed in all groups, except controls
		- Immunohistochemical determination of MMP-1, MMP-3, and TIMP-1 expression	- Cell apoptosis induced by MMP and TIMP-1 expression
		- TUNEL staining	- Weightlessness and hypergravity may both aggravate IDD
Johnston et al. (2010)	Risk for developing herniated NP after spaceflight	Incidence rates of HNP in astronauts using the Longitudinal Study of Astronaut Health database	- Incidence of HNP was 4.3 times higher in astronauts
			- More HNP in the cervical region of the spine
Laws et al. (2016)	Effects of swelling-induced disc height increases on spine, annular strain, and nuclear pressure during forward bending	Eight human lumbar motion segments subjected to baseline flexion and compression and pure moment flexibility tests	- Decrease in flexibility and increase in annular strain and nuclear pressure after the swelling-induced disc height increase
		- Free-swelling to vary supraphysiologic heights consistent with prolonged weightlessness	- Loss of lordotic curvature with disc swelling
			- Increased herniation risk
Bailey et al. (2018)	Quantitative measures of lumbar spine anatomy, health, and biomechanics in astronauts	Six NASA astronauts involved in a 6-month mission aboard the ISS	- Decrease in supine lumbar lordosis
		**-** 3T magnetic resonance imaging and dynamic fluoroscopy of the lumbar spine pre- and post-flight	- Decrease in active flexion–extension range of motion (FE ROM) for the middle three lumbar discs
			- No significant passive FE ROM changes
			- No difference in disc water content from pre- to post-flight
			- Changes in multifidus and erector spinae, correlated with lordosis, chronic low back pain, and disc herniation
Stamenković et al. (2010)	Deposition of cartilage-specific ECM components and cellular organization in microgravity	Porcine chondrocytes cultured in (i) microgravity during Flight 7S on the ISS, (ii) simulated microgravity in a random positioning machine (RPM), and (iii) normal gravity (1 g, control)	- Weaker ECM staining of ISS neocartilage tissues
		- Histology, immunohistochemistry, quantitative real-time PCR, and histomorphometric analysis	- Higher collagen II/I expression ratios in ISS samples
			- Higher aggrecan/versican gene expression profiles in control 1g
			- Reduced cell density in microgravity was significantly reduced compared with the normal gravity neocartilage tissues
Földes et al. (1996)	Structure and macromolecular composition of rat lumbar IVD during a 12.5-day spaceflight	Semiquantitative histochemical, topo-optical reactions were measured and evaluated by retardation values of birefringence	- Lateral expansion and accumulation of notochordal cells in the NP
			- Swelling of cartilage endplates, involving hypertrophied cells with mild extracellular mineralization

			- Mild decrease in orientation of collagen fibers in the external zone of AF and cartilage endplate
			- Increase in orientation of hyase-sensible glycosaminoglycans in the internal zone of AF and NP
			- Increase in orientation of glycoproteins in AF
Pedrini-Mille et al. (1992)	Composition of IVD and biomechanical functions of the AF	The lumbar annuli of rats flown on COSMOS 2044 were compared with those of three control groups and a tail-suspension experimental model	- Significant smaller wet and dry weights of AFs in the flight group
			- Greater collagen-to-proteoglycan ratio in the flight group
			- No detectable collagen or in the number of pyridinoline cross-links
			- Abnormal or smaller proteoglycans in flown rat AFs
			- Normal spatial distribution of the proteoglycans in AF
			- Tail suspension did not affect the size of the annuli but increased leached proteoglycans
Franco-Obregón et al. (2018)	Role of transient receptor potential canonical (TRPC) channels in IVD mechanosensing	Primary human IVD cells exposed to simulated microgravity and to the TRPC channel inhibitor SKF-96365 (SKF)	- Reduced proliferation and increased senescence after simulated microgravity and TRPC channel inhibition
		Proliferative capacity, cell cycle distribution, senescence, and TRPC channel expression	
			-Reduced TRPC6 expression with simulated microgravity

### 5.1 Microgravity *in vivo* analysis

Despite the evidence of the increased risk of IVD herniation for astronauts, the precise impact of microgravity on IVD and its association with IDD have not been studied in depth. Different studies reported the effects of microgravity on disc height, ECM degradation, AF fiber alignment, and NP swelling, suggesting an increased risk of herniation in cases of prolonged and repeated space missions. For this reason, both *in vivo* experiments and *in vitro* 2D and 3D cellular models have been exploited to recapitulate the impact of spaceflight conditions on the IVD of healthy astronauts (Smith and Mercuri, 2021).

An experiment conducted by Bailey et al. (2014) aimed to observe microgravity-induced changes in lumbar and caudal IVDs in the C57BL/C mouse model. The experimental mice models participated in the STS-131 space mission and were sacrificed immediately after spending 15 days in space. The researchers observed that in the caudal IVDs of mice subjected to spaceflight, there was a 32% decrease in IVD height and a 70% reduction in the sliding parameter given by nuclear bulge. For lumbar IVDs, no changes in either height or nuclear pressure were observed. Furthermore, microgravity did not affect either annular viscoelasticity or endplate permeability of the lumbar and caudal vertebrae. Given the difference in the results between the caudal and lumbar vertebrae, the authors stated that the absence of load and the continuous movement of the vertebrae could be the reason for the higher incidence of cervical disc herniation than that of lumbar disc herniation in astronauts.

A further study used 120 rabbits divided into control, microgravity (suspension through the tail), hypergravity (animal models subjected to a 1-min centrifuge at +7 G three times) and mixed groups. The animal models were subjected to these conditions for 30, 60, and 90 days. From the results, increased expression of metalloproteinases such as MMP-1, MMP-3, and TIMP-1, enzymes responsible for ECM degradation, was observed in the mixed group, followed by the hypergravity and microgravity groups, and a pattern of apoptosis activation was observed. With this study, the authors confirmed that both microgravity and hypergravity have a strong impact on the onset of IDD (60). The same group used the same subdivision of rabbit animal models by exposing them to microgravity and hypergravity for 4, 8, and 24 weeks. After exposure, the authors assessed the body weight of the animals, which increased in the control group alone and decreased in all other groups. In addition, the glycosaminoglycan (GAG) content in the three groups was significantly lower than that in the control group, suggesting that changes in gravity may be involved in the development of IDD (Wu et al., 2019).

A study on 321 astronauts showed that the risk of lumbar disc herniation after a space mission is 4.3 times higher in astronauts than in the general population (Chang et al., 2016). In agreement with this result, a NASA study states that the risk of cervical herniation in astronauts is 35.9 times higher than that in control and 2.8 times higher than that of lumbar disc herniation; moreover, the risk is higher in the first period after return to Earth (Johnston et al., 2010; Belavy et al., 2016). Surprisingly, the duration of spaceflight did not increase the risk of herniation occurrence due to the necessary precautions applied after spaceflight (Johnston et al., 2010). The cause of hernia in these cases may be attributed to physiological hydration of the IVD during exposure to microgravity burdening the AF, increasing the risk of hernia (Laws et al., 2016; Green and Scott, 2017). However, hydration is transient/not statistically significant for astronauts during spaceflight. The study also confirmed that spaceflight affects other pathophysiological features, such as a reduction in GAGs, increased collagen–proteoglycan ratio, and increased expression of metalloproteins in the ECM (Freed et al., 1997; Owen et al., 2020; Panesar et al., 2020).

A prospective longitudinal study on six volunteer astronauts evaluated any changes in the spine after a 6-month mission on the ISS. To obtain these results, 3T magnetic resonance imaging (MRI) and dynamic fluoroscopy of the spine were performed. These examinations were performed 30 days after takeoff and repeated the day after landing on Earth. The results obtained showed a flattening of lordosis with an average of 11% among the subjects. In the central lumbar IVDs (L2–L5), there was a decrease in the active flexion–extension range of motion, while the passive showed no change. In 20% of the subjects, there was a decrease in the mean functional cross-sectional area and a 8%–9% decrease in the cross-sectional area of the multifidus and spinal erector spinae. In addition, changes in multifidus were correlated with changes in lordosis. However, only two subjects with severe pre-flight irregularities had lumbar pain or hernia after flight. From this study, the authors showed that multifidus atrophy, as opposed to IVD swelling, is associated with lumbar flattening by increasing stiffness and, if present simultaneously with pre-flight vertebral endplate irregularities, exponentially increases the likelihood of the onset of disc disease (Bailey et al., 2018).

### 5.2 Effects of microgravity on cellular models

Studies on cell models placed under real or simulated microgravity conditions have shown important cellular and molecular changes in IVD, impacting structural composition and thus functionality. (Jin et al. 2013) proved that mouse IVDs cultured under microgravity conditions simulated by the RWV experienced a downregulation of GAG content and an upregulation of MMP-3 and apoptotic mediators, concluding that microgravity is able to mimic the *in vivo* degeneration events. The association between IDD and inflammation in microgravity condition has been confirmed by the altered expression levels of tumor suppressor proteins p53 and p16 in rat models. The authors reported a significant IVD injury observed by MRI in the rat experimental group. They also noted histological signs of IDD, upregulation of inflammatory factors (TNF-α, IL-1β, and IL-6), and expression of p53 and p16 both at mRNA and protein levels. Furthermore, a statistically significant correlation was observed between these variations and the degree of IDD (Li et al., 2019). From the structural point of view, an imbalance in the expression and production of proteoglycans present in the IVD demonstrated the alteration in structural composition. In fact, studies on the ratio of aggrecan (cartilage-specific proteoglycan) to versican (general proteoglycan) showed a lower ratio in porcine chondrocytes cultured aboard the ISS. Using quantitative polymerase chain reaction (PCR), Stamenković et al. (2010) revealed that ISS tissue displayed higher expression ratios of collagen type II to type I than normal gravity control tissue, despite not translating to higher protein levels. The author attributes this mechanism to a negative effect of microgravity on collagen post-translational modifications and potential readaptation to gravity upon return of the ISS sample.

In a study on GAGs conducted by Földes et al. (1996), rats were exposed to microgravity aboard the Cosmos 1887 biosatellite for 12.5 days. The rats subjected to spaceflight exhibited a different distribution of collagen in the outer AF and endplate of cartilage and also showed hypertrophy with mild mineralization. In the NP, notochordal cells were found with a predominant population of choroid cells. There was also a significant increase in GAG orientation in both the outer and inner areas of the AF and NP. All these features, according to the authors, could be molecular causes of many of the axial pathologies faced by astronauts.

Pedrini-Mille et al. (1992) analyzed the IVD of rats involved in the COSMOS 2044 mission. This study showed that there was a reduction in weight of rat AF compared to the control (−20%). Indeed, in the spaceflight cohort, the collagen/proteoglycan ratio was greater, but there was no significant difference in the proportion of collagen I or II. A further experiment, conducted by submerging the AFs in water to let the proteoglycan diffuse, showed that a greater amount of proteoglycans was released from the AFs subjected to spaceflight than that from the control subjects. The authors associated the increased loss of proteoglycans with an abnormal conformation of proteoglycans or a reduction in the volume of the molecule, stating that these changes may affect the biomechanics of the AF.

Recently, Doty et al. (1999) studied how spaceflight could alter cell cycle, differentiation, apoptosis, and proliferation processes. For this study, chick embryonic mesenchymal cells were divided into two populations: a control group (1 G) and a spaceflight group aboard STS-95 for 9 days. Flow cytometry revealed that cells subjected to spaceflight showed higher expression of cyclin E, proliferating cell nuclear antigen (PCNA), and p27 and a lower G1 phase of the cell cycle than the control group. Analysis of cell culture media showed that spaceflight cells continuously metabolized glucose to lactate during the 9 days of spaceflight. This analysis affirms how spaceflight affects the cell cycle but not apoptosis, which can also be confirmed by the presence of cells in the G1 phase of the cell cycle.

Franco-Obregón et al. (2018) conducted studies on cells isolated from AF and NP biopsies and showed that treatment with the TRPC-SKF-96365 (SKF) channel inhibitor for up to 5 days under microgravity conditions resulted in a reduction in proliferative capacity and a consequent increase in disc cell senescence. Furthermore, treatment with SKF channel inhibitor led to a change in the cell cycle of disc cells by increasing the G2/M phase, thus affirming that induced microgravity has consequences on cell cycle and cell senescence.

### 5.3 Microgravity and therapeutic approaches

Several experimental and clinical studies are currently underway to investigate the benefits that microgravity can provide to individuals suffering from acute injuries or chronic diseases of the musculoskeletal system.

As mentioned, immersion is a technique used to simulate microgravity and its effects, including the reduction in load on the musculoskeletal system that allows individuals with musculoskeletal disorders to obtain relief (Bender et al., 2005). In addition, the water heating technique raises patients’ body temperature, reduces gamma fiber activity of motor neurons, and reduces activity and spasticity. All these contribute to better muscle-articular alignment, which allows the range of motion to be expanded, improving the subjects’ mobility and increasing the effectiveness of this rehabilitation technique (Bellomo et al., 2012). The analgesic effect of this technique can be attributed to its action on mechanical and thermal receptors and the blocking of pain perception (Lange et al., 2006). It has also been shown that patients undergoing this rehabilitation can reduce the intake of drugs, thus avoiding their side effects (Yalcinkaya et al., 2014).

## 6 Conclusion

The studies analyzed were undertaken with the aim of identifying the relationship between microgravity, understood as a peculiar condition to which astronauts are subjected, and IVD degeneration, which can result in a pathological condition that can affect the performance of astronauts themselves, limiting their operations. This overview described several studies revealing the effective role of microgravity, and space conditions in general, in IVD pathophysiology, in terms of structural and functional modifications, as well as the onset of an inflammatory microenvironment. Taken together, these findings suggest the necessity to better investigate the associations between these two mechanisms in order to identify the molecular mechanisms characterizing IDD and to apply specific preventive countermeasures to safeguard the health of pilots and ensure or increase their work efficiency and performance. In addition to being a major ailment afflicting pilots and astronauts, it should be noted that IDD, as well as associated LBP, is one of the most disabling causes of the world’s population, so the recognition of novel cellular and molecular targets and potential preventive approaches could have a potent impact on civilian and military healthcare costs.
